# Does farmer entrepreneurship alleviate rural poverty in China? Evidence from Guangxi Province

**DOI:** 10.1371/journal.pone.0194912

**Published:** 2018-03-29

**Authors:** Eric Yaw Naminse, Jincai Zhuang

**Affiliations:** 1 School of Management, Jiangsu University, Zhenjiang, PR China; 2 School of Economics and Management, Qinzhou University, Qinzhou, PR China; University of West London, UNITED KINGDOM

## Abstract

In recent years, entrepreneurship has been gaining more prominence as a potential tool for solving poverty in developing countries. This paper mainly examines the relationship between farmer entrepreneurship and rural poverty alleviation in China by assessing the contribution of farm entrepreneurs towards overcoming poverty. Data were collected from 309 employees of farmer entrepreneurships in Guangxi Province through survey questionnaires. Structural equation modeling was used to conduct an analysis of the effects of three identified capabilities of farm entrepreneurs—economic, educational and knowledge, and socio-cultural capabilities—on attitude towards farmer entrepreneurship growth and the qualitative growth of farmer entrepreneurship and how these in turn affect rural poverty, using AMOS 21. The findings show that socio-cultural capability has the greatest influence on farmer entrepreneurship growth (β = 0.50, p<0.001). The qualitative growth of farmer entrepreneurship also more significantly impacts rural poverty (β = 0.69, p<0.001) than attitude towards farmer entrepreneurship growth. This study suggests that policy makers in China should involve more rural farmers in the targeted poverty alleviation strategies of the government by equipping rural farmers with entrepreneurial skills. This can serve as a sustainable, bottom-up approach to alleviating rural poverty in remote areas of the country. The study also extends the literature on the farmer entrepreneurship-rural poverty alleviation nexus in China, and this can serve as a lesson for other developing countries in the fight against rural poverty.

## Introduction

Since the turn of the 21^st^ century, one of the major challenges facing most emerging and transitional economies, including China, is poverty. Evidence shows that many of the world’s poorest people reside in rural areas and subsist on an income of less than US$1.25/day with agriculture and forest activities serving as their dominant economic choices. Rural poverty currently remains the predominant form of human deprivation in the world and affects many lives in both the developed and developing worlds.

In China, the unprecedented economic growth, averaging 10% per annum in the last 35 years [1], led to over 700 million people being lifted out of poverty. However, more than 70 million people still live below the national poverty line of US$ 354/year or its equivalent of 2,300 RMB per year (by 2010 price standards) [2]. This is evidence that the incidence of rural poverty in China is rising in spite of the country’s economic success in recent years. Some programs instituted in the past to fight against rural poverty yielded low results. For instance, the “Go West” program led by China’s central government in support of rural development. Although it yielded some positive results through increases in the supply of rural public goods and high technology transfers in many western parts of the country, it failed to improve people’s overall living standards, especially rural residents [3]. It has been estimated that about half of China’s rural poor live in the western provinces compared with 36% and 14% in the central and eastern parts respectively [4]. It is also estimated that the rural poverty rate in the western parts of China is approximately 5.7%, which is higher than the 2.8% in central and 1% in eastern parts of the country. This has attracted serious national concern from the government and other policy makers regarding how to curb the problem in order to prevent conflicts, social unrest and political instability. The lack of quality human capital in rural areas is often blamed for the low incomes associated with rural people, most of whom are farmers. They often lack the skills to take advantage of economic opportunities [5] to improve their living conditions. Meanwhile, since Schumpeter placed entrepreneurship at the center of economic growth, many studies have shown that entrepreneurship plays an important role in stimulating economic growth [6]. Moreover, there has been a recent increase in studies which show that entrepreneurship has the potential to reduce poverty and conflicts in developing countries [7,8].

However, few empirical studies exist on the role of farmer entrepreneurship in rural poverty alleviation. Thus, this paper explores the relationship between farmer entrepreneurship and rural poverty alleviation in Guangxi Province of China using the capability approach [9].

In this study, we define farmer entrepreneurship as both farm and non-farm activities undertaken by individuals for profitable gains [10]. In other words, a farm entrepreneur is an individual employed either on a full time or part-time basis in farming activities, such as soil cultivation, crop growing, and livestock rearing, as the principal source of their income. This is critical because, in China, agriculture still employs approximately 64% of the population, and efforts to alleviate rural poverty would require attention to farmers. The study will therefore help policy makers in China to address rural poverty from farmers’ own perspective. The study is also consistent with the main goal of the 13th Five-Year Plan (2016–2020) of the Chinese government to prioritize rural poverty alleviation as part of the long-term social, economic and cultural development of the country.

The remaining structure of the paper is as follows: A brief explanation on rural poverty and farmer entrepreneurship and the relationship between them, theory and hypotheses development, materials and methods, results and discussion, and conclusions.

### Understanding rural poverty

The concept of poverty is said to be multidimensional in nature and, thus, has various meanings. Poverty is defined as the lack of necessary resources to permit people’s participation in activities, customs and diets commonly approved by society [11]. In an attempt to emphasize this definition, the European Commission [12] also defines people in poverty as those whose incomes and/or resources are so inadequate as to preclude them from having what society considers an acceptable living standard. One of the most widely used definitions is by the World Bank [13], which defines poverty as the pronounced deprivation of human well-being based on four dimensions, namely, (a) a lack of basic human necessities (food, water, shelter, and clothing), (b) a lack of access to basic education; (c) a lack of primary healthcare; and (d) a lack of security and protection against discrimination. Poverty is also defined in absolute and relative terms [14,15], while other scholars have defined poverty based on income and multidimensionality [16–18] as well as whether it is transient or chronic [19,20] or rural or urban in nature [21–23].

From the above, it is clear that income alone cannot be used to measure poverty because poverty is a complex and dynamic phenomenon [24]. Rural poverty is defined in this work to include the lack of economic, social, and cultural capabilities of individuals from commanding the minimum standard of living based on Sen’s [9] capability approach. While rural poverty in developing countries is linked with food insecurity, rural poverty in China has to do with limitations in human skills and competencies, which prevent people from taking advantage of available economic opportunities to create wealth for themselves and their societies. Rural poverty is also defined as human deprivation, which occurs in non-metropolitan areas with populations below 50,000 and where there are more single-guardian households, less access to public services and support for disabilities, and limited education and healthcare opportunities. Thus, in this paper, rural poverty is regarded as the lack of economic, socio-cultural and educational capabilities of farmers to be able to convert opportunities into profitable business ventures to improve their living conditions.

### Understanding farmer entrepreneurship

Entrepreneurship is widely seen as a driver of economic growth in developed and developing counties [25]. Entrepreneurship is viewed not only as a multidimensional concept but as a dynamic academic field [6] with varied meanings. For instance, entrepreneurship is often defined as the process of ‘creative destruction’ by innovative individuals in an economy [26]. It also refers to the conversion of existing opportunities to create future goods and services [27].

However, the 2014 Global Entrepreneurship Monitoring (GEM) report [28], defines entrepreneurship as any attempt at a new business or new venture creation, such as self-employment, new business organization, or the expansion of an existing business by an individual, a team of individuals or an established business. Farmer entrepreneurship, on the other hand, is defined as a farm and/or non-farm activity undertaken by persons either on a full time or part-time basis to command an earning [10], where the farming activity involves soil cultivation, crop growing, and livestock rearing as the main source of income.

Farmer entrepreneurship is also related to farm diversification, self-employment or agribusiness related activity in which a greater degree of autonomy is exhibited in controlling, organizing, and management risks and resources to achieve higher gains. It is argued that the desire to increase household income has been the key motivating factor that drives farmers to become entrepreneurs in recent times.

### Relationship between farmer entrepreneurship and rural poverty

A plethora of literature has examined the issue of entrepreneurship and poverty in developing countries [29–33] but not specifically the relationship between farmer entrepreneurship and rural poverty. For instance, using time series data from China, it is found that agriculture contributed immensely to poverty reduction in the 1980s through increased farm productivity [34]. Another investigation, on how the rural ‘*dibao*’ (minimum living standard guarantee) program in China impacted poverty, found that the pro-poor program enabled beneficiary households to overcome rural poverty [35].

The effects of location and sectoral components of economic growth on poverty in Indonesia was also examined, and the results show that rural agricultural growth greatly impacted poverty reduction [36]. In sub-Saharan Africa, for instance, the effects of agribusiness on poverty revealed that agriculture entrepreneurial growth impacted significantly on poverty [37]. In using the capability approach to determine how the rural poor can escape from poverty, a study found that age, gender, marital status, economic activity, and healthcare were key determinants of community and individual economic empowerment through which people’s capabilities helped to overcome poverty [38]. From the above, it appears there is a relationship between entrepreneurship and poverty. However, no or few studies have explored the link between farmer entrepreneurship and rural poverty in China, which has a relatively high rural population of approximately 46.27% with most of the poor located in rural areas in Guangxi Province [39].

The potential innovations of this paper are as follows. First, few studies have explored the link between farmer entrepreneurship and rural poverty alleviation in China using structural equation modeling. Hence, this paper will help lay a foundation for building theory and practice in the entrepreneurship and poverty literatures in developing countries. Second, the paper will deepen the understanding of how farm entrepreneurs’ capabilities can help to improve the growth of farmer entrepreneurship and alleviate rural poverty. Finally, by combining structural equation modeling (SEM) with the capability approach, the paper presents a unique opportunity to extend the empirics of farmer entrepreneurship and rural poverty alleviation studies.

### Theory and hypotheses development

The development of entrepreneurial skills is necessary for farmers if they are to succeed in their farm businesses. It is even more important when opportunities are located in rural communities, which lack institutional support [40]. A search for an effective match between farm entrepreneurial skills and market opportunities to build up the competitive urge of entrepreneurs in farming, therefore, requires critical examination. This is important because entrepreneur competence is needed for the development of new enterprises [41]. Thus, this paper employs the capability approach [42,43] to examine how three identified capabilities of farm entrepreneurs, namely, economic, educational and knowledge, and socio-cultural capabilities, influence farmer entrepreneurship growth and rural poverty alleviation in China.

### The capability approach (CA)

The capability approach is an approach developed by Amartya Sen that is used in human wellbeing assessment [44]. Human capital development in the form of education is usually considered an effective tool for successful entrepreneurship and poverty alleviation. Therefore, capabilities required to alleviate rural poverty are associated with farm entrepreneurial activities that are undertaken. The capability approach is based on the notion that poverty is a multidimensional concept, and the development of human skills is needed for its sustainable alleviation [9,28]. Hence, the capability approach is used to measure human wellbeing from the perspective of giving people freedom through the expansion of their capabilities. The approach focuses on the functioning or living conditions of individuals, which are defined as what people can or cannot do or what they can or cannot be [9,44]. It is more concerned with the ability or capacity of persons to achieve freedom of development [42]. Although some scholars have criticized the approach for lacking specific indicators to measure poverty, others have lauded it and provided indicators for measuring human wellbeing [45,46]. According to the literature on the capability approach, there are four components: commodities (goods or services that an individual or household declares are legally obtained in using an endowment), functioning (the achievements, that is, the doings or beings of a person), utility (the desired fulfillment of an individual) and endowment. The capability approach is perhaps one of the most appropriate ways to address such potentially multidimensional issues as poverty. Nussbaum [43] argues that the capability approach begins with normative assumptions that all people have equal dignity and that they should all enjoy the capacity for a life of equal dignity. For Nussbaum, such a goal has to be sensitive to the complexity of the interdependent relations that support people’s living conditions.

### Study hypotheses

In many developing countries, human skills are widely lacking, and in many cases, people with higher skills remove out of agriculture to other sectors of the economy [47]. This makes it difficult for the governments of such countries to retain skillful farmers for entrepreneurial activities. However, the acquisition of entrepreneurial skills by rural farmers can help increase productivity and enhance incomes and quality of life through greater confidence [48,49]. Entrepreneurial opportunities can also easily be identified when people’s capabilities are improved, and this can lead to reductions in poverty [50]. In this study, three capabilities of farm entrepreneurs, namely, economic (measure of children’s education, village schools’ conditions, income levels), educational and knowledge (access to information and knowledge), and socio-cultural capabilities (an openness to decision-making processes and social networking), are used for the investigation.

### Link between farm entrepreneurs’ economic capabilities and farmer entrepreneurship growth

The economic capabilities of farm entrepreneurs, in this paper, includes farm income levels, access to market information, and chances to acquire and use new farm technologies, as well as children’s access to higher education. In studies by Koch [51] and Banerjee et al. [52], they found that growth in agricultural income can reduce poverty. Therefore, the ability of farm entrepreneurs to increase farm and non-farm earnings is dependent on their economic capability levels. The ability of children from farm households to access quality and higher education can be a measure of the economic capabilities of farm entrepreneurs. Weaver et al. [53] investigates the influence of four capabilities on employees at different stages of the innovation process, using data from 264 surveys in China, and realized that firms typically adopt management innovations, which are facilitated by socio-economic capabilities, to improve the firm’s performance. The capability approach has also been widely applied in health economics to measure the wellbeing of individuals [54]. It is therefore expected that farmers’ incomes can be reinvested in non-farm businesses to create jobs, which promotes the growth of entrepreneurship, which can lead to poverty reduction. Therefore, we propose that

**H1a.** There is a positive effect of farm entrepreneurs’ economic capabilities on the attitude towards farmer entrepreneurship growth.
**H1b.** There is a positive effect of farm entrepreneurs’ economic capabilities on the qualitative growth of farmer entrepreneurships.

### Link between farm entrepreneurs’ educational and knowledge capabilities and farmer entrepreneurship growth

Knowledge can be gained through the education and training of individuals. In Uganda, it has been found that education has a positive impact on rural poverty [55]. In China, education helps to improve both farm and non-farm incomes, but the level of return from education in the western areas is still low, ranging from 2.7 to 3.9% [56]. Furthermore, it has been found that rural residents engaged in off-farm employment in China are less likely to migrate to big cities [57]. Education facilitates the accumulation of human capital for development, and it can also lead to improvements in the entrepreneurial exploits of individuals [58]. It has also been found that the level of people’s education and their welfare are positively correlated [59,60]. Since the quality of education is mostly low in rural China, and children from farming backgrounds usually drop out of school early, it seems efforts to alleviate rural poverty should consider improving education in order to enhance the capabilities of farm households to improve living conditions. Based on the above, we hypothesize that

**H2a.** There is a positive effect of farm entrepreneurs’ educational and knowledge capabilities on attitudes towards farmer entrepreneurship growth.
**H2b.** There is a positive effect of farm entrepreneurs’ educational and knowledge capabilities on the qualitative growth of farmer entrepreneurship.

### Link between farm entrepreneurs’ socio-cultural capabilities and farmer entrepreneurship growth

Social and cultural competences are interrelated, and they form part of individuals’ abilities to attain higher business goals. Social capital and cultural orientations tend to affect poverty alleviation strategies in society [61]. Culture, on the other hand, is the deposit of knowledge, experience, beliefs, values, attitudes, meanings, hierarchies, religion, notions of time, roles, spatial relations, concepts of the universe and material objectives and possessions acquired by a group of people over the course of generations through individual and group striving [62]. Culture is dynamic and helps to shape how people in society interact with one another, either collectively or individually. Culture has an impact on poverty and how it can be alleviated in a society. Social skills refer to the relations or ties that exist among people [63]. These social relations and culture are embedded in business decision-making processes and are often referred to as ‘*guanxi*’ in China [64]. The term expresses trust in relationships among families, friends, government, and communities, as well as business partners that seek resources, information and support for individuals in the growth of businesses [65,66].

The socio-cultural capabilities of farm entrepreneurs comprise social interactions, networking abilities, improved culture, and available opportunities to farmers. Using 293 creative entrepreneurs in China, Chen et al. [67] found that socio-cultural attributes positively influence entrepreneurial activities. The socio-emotional competencies of employees in American and South Korean firms was also assessed using the regression model, and it was found that employees usually seek a balance between social and cultural competencies, which tends to increase their performance in organizations [68]. A study on the relationship between organizational justice and outcomes in India found that trust partially mediated an increase in performance [69]. Thus, the socio-cultural capabilities of farm entrepreneurs can affect the growth of entrepreneurship. We therefore, hypothesize that

**H3a.** There is a positive effect of farm entrepreneurs’ socio-cultural capabilities on attitudes towards farmer entrepreneurship growth.
**H3a.** There is a positive effect of farm entrepreneurs’ socio-cultural capabilities on the qualitative growth of farmer entrepreneurship.

### Link between farmer entrepreneurship growth and poverty alleviation

Entrepreneurial activities in China are widespread and the phenomenon is attributed to the expansion of rural industries in the early years of the 1978 rural reforms. A study on the contribution of township and village enterprises (TVEs) in China shows that a higher GDP growth rate resulted from the higher earnings of entrepreneurs [70]. The growth of farm enterprises can partly be attributed to attitude towards entrepreneurship [71] and the willingness of individuals to share their resources to benefit others in society (qualitative growth). The qualitative growth of farmer entrepreneurship is mostly evident in the provision of public goods and services in rural communities by individuals of the community. Attitude towards farmer entrepreneurship growth refers to the ability of farmers to found enterprises. People differ in their ways of starting up businesses. With the accumulation of skills and interpersonal networks, the attitude of farmers towards founding enterprises can be different. In rural Rwanda, attitude towards entrepreneurship growth was used to analyze poverty and livelihood profiles in a post-conflict situation. It was found that rural poverty was reduced through the combined factors of the natural, physical, human, financial and social resources and skills of farm households [72]. In Brazil, using a growth modeling approach, it was found that entrepreneurial skills training significantly increased organizational performance in nascent firms [73]. In the Henan Province of China, community-based land helped to support agricultural villages, leading to improvements in local living conditions [74]. It is therefore estimated that as more farmers become entrepreneurs in rural China the supply of public goods and services can be increased through voluntary contributions to reduce poverty. From the above, we hypothesize that

**H4a.** There is a positive effect of attitude towards farmer entrepreneurship on rural poverty alleviation in China.
**H4b.** There is a positive effect of the qualitative growth of farmer entrepreneurship on rural poverty alleviation in China.

## Materials and methods

### Ethics statement

This study was conducted under the research permit JU15/2346 approved on March 22, 2015, by the Ethics Committee of Jiangsu University in Zhenjiang City in the People’s Republic of China. Additionally, the consent of parents was obtained orally before interviewing respondents under 18 years old.

### Study areas

Three rural communities, namely, Baise, Liuzhou and Guilin in Guangxi Province, were purposely chosen for the study because they are regarded as areas with high rates of poverty in western China [75–78].

In 2008, for instance, the annual average farm income in the province was about US$ 387.21 person/year, which is close to China’s national income poverty line of US$ 377.01 person/year. Guangxi Province is officially called Guangxi Zhuang Autonomous region, and the ethnic minority and language spoken is Zhuang. The selected areas are suitable for the cultivation of tropical and subtropical fruits crops, such as garden tea, organic mango, star-fruit, gourd plant (*Cucurbitaceae*) and banana, as well as the rearing of sheep, goats and birds, such as ducks and chickens. The annual average temperature and rainfall range between 17°C-23°C and 1000 mm-2800 mm respectively [79].

### Survey instrument

The data were collected between July and August 2015. A semi-structured questionnaire measured on a 5-point Likert scale (1, strongly disagree; 2, disagree; 3, neutral; 4, agree; and 5, strongly agree) was used to assess the quality of rural life of 309 respondents [80]. Details of the survey questions are in S1 Table.

### Sample size and data collection

When using structural equation modeling (SEM), a higher sample size is preferred [81]. However, it has been found that a minimum sample size of 200 is acceptable in SEM analysis [82]. Hence, using multistage sampling technique, this paper employs 309 respondents, comprising eight communities in Baise, six in Liuzhou and two in Guilin, through random sampling (S2 Table). Researchers, after randomly picking these communities, chose twenty households from the communities and interviewed mainly employees of farmer entrepreneurships. Close contact with agricultural extension workers and dealing with communities where farmer entrepreneurship is operational enabled us to choose the respondents. The retrieval rate of the administered questionnaire is 86% (S2 Table).

### Method of analysis

#### Confirmatory factor analysis

An exploratory factor analysis was first performed using SPSS version 20 to determine the suitability of the data for factor analysis [82]. It was found that almost all the items had significant factor loadings except items 11 (0.381) and 14 (0.375), which were dropped because of their poor factor loadings. A confirmatory factor analysis (CFA) was conducted using AMOS version 21. Structural equation modeling (SEM) was chosen for the study due to its ability to mitigate problems of measurement errors in relationship studies involving latent variables. The SEM model is a two-part model, consisting of the measurement and structural models. The measurement model measures the relation between the observed and unobserved variables by providing a link between the scores on the measurement instrument. The structural model, on the other hand, measures the relationship among the unobserved variables by specifying the manner in which a particular latent variable either directly or indirectly influences or causes a change in the values of the other latent variables in the model.

The following statistical indices are used to assess the goodness of fit of the model based on their unique criteria [83–85]: chi-square (χ^2^), probability value (P), goodness-of-fit index (GFI), adjusted goodness-of-fit index (AGFI), root mean square error of approximation (RMSEA), normed fit index (NFI), comparative fit index (CFI), composite reliability (CR), and average variance extracted (AVE).

## Results and discussion

### Demographic profile of respondents

The demographic profile of respondents is given in detail in Table 1. The results indicate that 60.84% are male and 39.16% female. This implies that farmer entrepreneurship is a male-dominated sector, although women are often observed to be more involved in downstream agricultural activities in developing countries. This finding is in line with earlier research which found that women are usually disadvantaged in agriculture due to uneven access to land, especially in Asian countries [86,87].

**Table 1:** Respondents’ demographic profile.

Variable	Description	Frequency	Percentage (%)
Age	16–25	34	11.00
	26–35	78	25.24
	36–45	72	23.30
	46–55	79	25.57
	56–65	34	11.00
	66–75	9	2.92
	76–85	3	0.97
Gender	Male	188	60.84
	Female	121	39.16
Education level	Primary or below	65	21.04
	JHS	169	54.69
	SHS/Technical	63	20.39
	College and above	12	3.88
Marital status	Married	268	86.73
	Single	39	12.63
	Divorced	1	0.32
	Widowed	1	0.32

**Source:** Field data, 2015. N = 309, JHS = Junior High School, SHS = Senior High School

Furthermore, 25.57% of the farm entrepreneurs are aged from 46–55 years. However, the combined age groups of 26–35 and 36–45 constitute the largest percentage, 48.54%, which is a relatively young age level. Having many young Chinese engaged in farmer entrepreneurship means that rural-urban migration can be reduced. It also means the future of China’s agriculture is bright as in other developing countries where the youth is actively involved [88]. Married respondents make up 86.73%, while unmarried is 13.26%. This might be due to the fact that agriculture is labor-intensive and may require more labor from family members. Respondents’ educational level indicates that 21.0% have a primary education or less, 54.69% had education up to junior high level, 20.39% had up to the senior high and/or technical level, and only 3.88% reached college level. This indicates that access to education in China has improved at the basic level, but much needs to be done at college level. With an improved level of education, farm entrepreneurs’ skills and knowledge can enhance their farm activities through the adoption of new farming techniques [89].

### Descriptive statistics and correlation results

The mean, standard deviation, Cronbach’s alpha coefficient and factor loading results are displayed in Table 2.

**Table 2:** Descriptive statistics.

Construct	Item			CA(α)	Factor Loading
		Mean	SD		
ATFE	b1	3.85	1.14	0.90	0.88
	b2	3.52	1.28		0.86
FEQG	b3	2.67	1.28	0.74	0.83
	b4	3.33	1.13		0.96
EC	b5	3.84	1.16	0.80	0.76
	b6	3.85	1.07		0.84
	b7	3.89	1.09		0.73
EKC	b8	3.15	1.21	0.89	0.71
	b9	3.13	1.18		0.91
	b10	2.89	1.26		0.84
SCC	b16	3.25	1.14	0.84	0.85
	b17	3.26	1.13		0.82
	b18	3.48	1.08		0.89
RP	b12	3.28	1.08	0.83	0.73
	b13	3.19	1.05		0.79
	b15	3.60	0.98		0.85
	b19	3.71	1.07		0.70

Note: ATFE = attitude towards farmer entrepreneurship growth; FEQG = farmer entrepreneurship qualitative growth; EC = economic capabilities; EKC = educational and knowledge capabilities; SCC = socio-cultural capabilities; RP = rural poverty; SD = standard deviation; CA = Cronbach’s alpha (α). N = 309.

The Cronbach’s alpha values, which measure the relationship between the variables and their constructs [90,91], are relatively higher, and this indicates a good reliability of the constructs. All the Cronbach’s alpha values range from 0.74 to 0.90, which is above the minimum acceptable value of 0.70 [92]. The results of the correlation matrix analysis are reported in S3 Table.

### The measurement model

The overall fitness of the model is evaluated using the results of the measurement model [93] in Table 3. The results show how the average variance extracted (AVE) obtained from the factor analysis is used to measure the convergent validity or the common medium variance of all the constructs. According to the table, all the constructs have values greater than the threshold of 0.5 [84]. The composite reliability (CR) index, which measures the internal consistency of the constructs, also shows values ranged from 0.73 to 0.91, which exceed the recommended value of 0.7 [94]. In addition, the fit indices of the model (Table 3) are as follows: χ^2^/df = 3.74, GFI = 0.92, AGFI = 0.85, RMSEA = 0.05, CFI = 0.93, and NFI = 0.91 for the economic, educational and knowledge, and the socio-cultural capability constructs, while, in the case of attitude towards farmer entrepreneurship and the qualitative growth of farmer entrepreneurship, the fit indices are as follows: χ^2^ /df = 3.63, GFI = 0.93, AGFI = 0.84, RMSEA = 0.03, CFI = 0.89, and NFI = 0.94, and for the rural poverty (RP) construct, the fit indices are χ^2^ /df = 3.84, GFI = 0.87, AGFI = 0.81, RMSEA = 0.04, CFI = 0.88, and NFI = 0.90.

**Table 3:** Measurement model.

Item	Construct	S.E.	p-value	CR	AVE	χ2/df	GFI	AGFI	RMSEA	CFI	NFI
y5	←EC	0.20	0.000***	0.81	0.69	3.41	0.84	0.73	0.04	0.83	0.87
y6	←EC	0.20	0.000***								
y7	←EC	0.20	0.000***								
y8	←EKC	0.35	0.000***	0.89	0.72	3.74	0.92	0.85	0.05	0.93	0.91
y9	←EKC	0.35	0.000***								
y10	←EKC	0.35	0.000***								
y16	←SCC	0.28	0.000***	0.85	0.66	3.64	0.88	0.71	0.05	0.85	0.82
y17	←SCC	0.28	0.000***								
y18	←SCC	0.28	0.000***								
y1	←ATFE	0.39	0.000***		0.83	3.65	0.93	0.84	0.03	0.89	0.94
y2	←ATFE	0.39	0.000***								
y3	←FEQG	0.23	0.000***	0.73	0.68	3.57	0.75	0.89	0.05	0.76	0.91
y4	←FEQG	0.23	0.000***								
y12	←RP	0.34	0.000***	0.84	0.58	3.84	0.87	0.81	0.04	0.88	0.90
y13	←RP	0.34	0.000***								
y15	←RP	0.41	0.000***								
y19	←RP	0.46	0.000***								

Cut-off Criteria: CR ≥0.07; AVE>0.05; χ2/df<5; GFI>0.90; AGFI>0.90; RMSEA<0.08; CFI >0.90; NFI>0.90; CA≥0.5. Note:

*p<0.05;

**p<0.01;

***p<0.001.

Previous studies [95,96] argue that fit indices, such as the GFI, AGFI, CFI, and NFI, must be greater than or equal to 0.90, while other researchers [69,97] suggest that RMSEA = 0.06, and GFI = 0.85 indicate good fitness of the model. Therefore, the results show that the model fits the data well.

### The structural model

**Fig 1:**
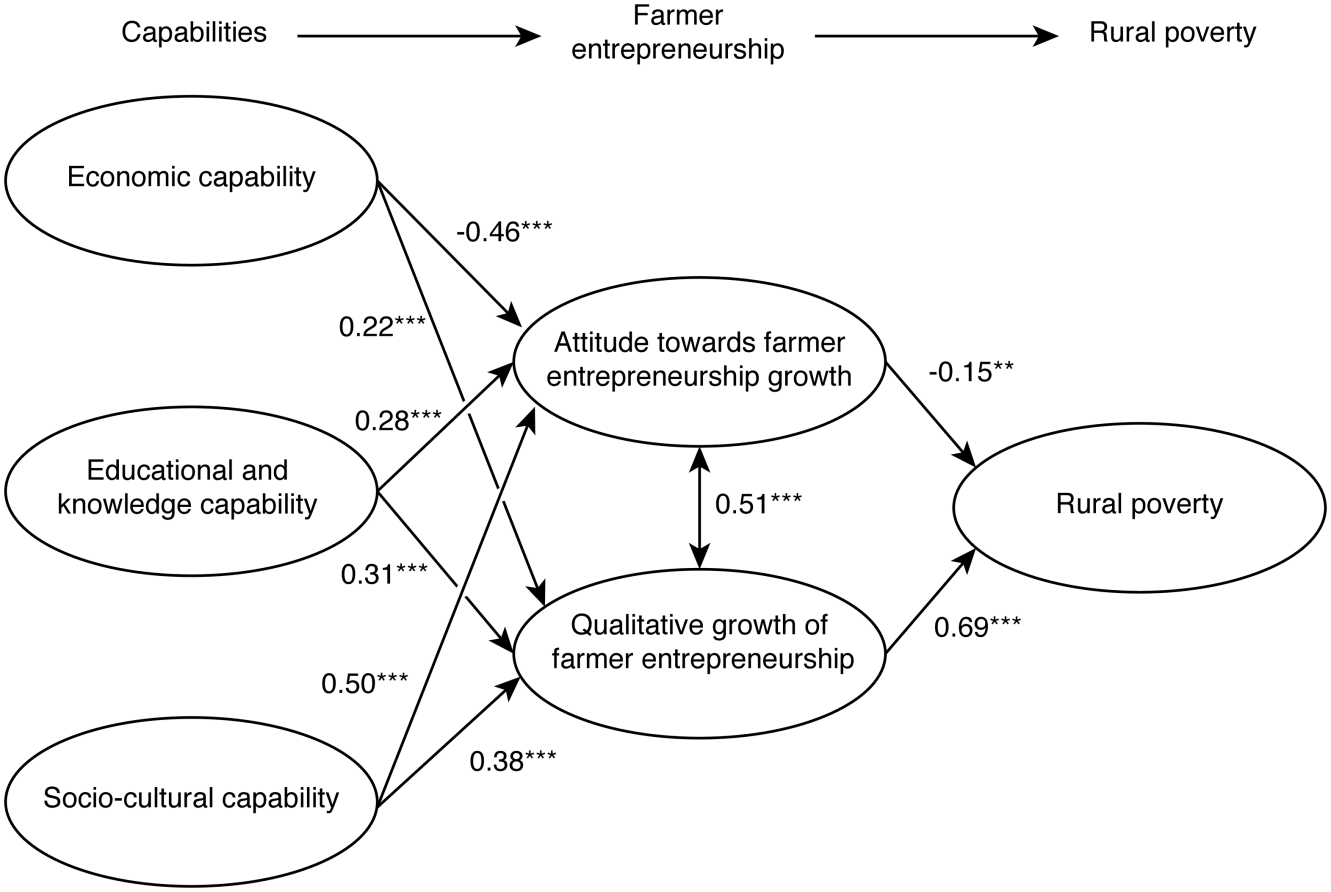
Structural equation model with standardized path coefficients.

For direct measurement of the relationship between the latent variables using standardized estimates, as shown in Fig 1, all except the economic capabilities have a positive and significant relationship with attitude towards farmer entrepreneurship and the qualitative growth of farmer entrepreneurship.

It can be seen that only the relationship between economic capabilities and attitude towards farmer entrepreneurship growth is negative, although it is still significant (β = -0.46). It can also be observed that socio-cultural capabilities have the greatest positive effect on attitude towards farmer entrepreneurship growth (β = 0.50) and the qualitative growth of farmer entrepreneurship (β = 0.38). This implies that although the economic, educational and knowledge capabilities of farm entrepreneurs are important, social-cultural capabilities tend to have a greater effect on the growth of farm entrepreneurial activities. With regard to the direct effect of attitude towards farmer entrepreneurship and the qualitative growth of farmer entrepreneurship on rural poverty alleviation, it is the latter that has the stronger effect (β = 0.69), while the former has a negative but significant effect (β = -0.15) on rural poverty. This implies that in order to alleviate rural poverty it is imperative to emphasize the need to promote the qualitative growth of farmer entrepreneurship.

### Hypothesis testing

The tests of hypotheses regarding the three capabilities of farm entrepreneurs, namely, economic capabilities (EC), educational and knowledge capabilities (EKC), and socio-cultural capabilities (SCC) are shown in Table 4 below.

**Table 4:** Hypotheses testing.

Path of Hypothesis	Estimate (β)	t-value	P-value	Hypothesis supported/Not supported
H1a: EC→ATFE	-0.46	-9.31	0.000***	Not Supported
H1b: EC→FEQG	0.23	2.42	0.000***	Supported
H2a: EKC→ATFE	0.28	2.84	0.000***	Supported
H2b: EKC→FEQG	0.31	2.92	0.000***	Supported
H3a: SCC→ATFE	0.50	6.15	0.000***	Supported
H3b: SCC→FEQG	0.38	4.93	0.000***	Supported
H4a: ATFE→RP	-0.15	-0.95	0.000**	Not Supported
H4b: FEQG→RP	0.69	7.32	0.000***	Supported

Note:

*p<0.05;

**p<0.01;

***p<0.001

Based on Table 4 above, it can be seen that except for the effect of the economic capability (EC) of farm entrepreneurs on attitude towards farmer entrepreneurship (ATFE), which is negative but significant, all other variables have shown direct significant and positive effects on attitude towards farmer entrepreneurship growth and the qualitative growth of farmer entrepreneurship. Further still, it is evident that the qualitative growth of farmer entrepreneurship (FEQG) has a significant positive effect on rural poverty (RP), while attitudes towards farmer entrepreneurship growth (ATFE) has a significant but negative effect on rural poverty (RP). This indicates that the qualitative growth of farmer entrepreneurship has a greater effect (β = 0.690, p <0.001) on rural poverty than attitude towards farmer entrepreneurship. Therefore, the following hypotheses: H1b, H2a, H2b, H3a, H3b and H4b are supported by the study, while hypotheses H1a and H4a are not supported.

## Conclusions

This study assesses the relationship between farmer entrepreneurship and rural poverty alleviation in the Guangxi Province of China. Using a survey sample size of 309, the paper employed structural equation modeling with AMOS 21, alongside SPSS, to conduct the analysis which tested the relationship between farmer entrepreneurship and rural poverty alleviation in China. The mediating effect of changes in the perception towards farmer entrepreneurship among rural people is also examined. Three capabilities of farm entrepreneurs—economic capabilities (EC), educational and knowledge capabilities (EKC) and socio-cultural capabilities (SCC) were used to measure the quality of life of rural respondents on a 5-point Likert scale. The findings indicate that socio-cultural capabilities have the greatest effect on attitude towards farmer entrepreneurship growth (β = 0.50) as well as on the qualitative growth of farmer entrepreneurship (β = 0.38), and this finding is supported by Chen et al. [67].

This is followed by the effects of educational and knowledge capabilities, which was also found by Sanz et al. [60], and then economic capabilities have the least effect. The qualitative growth of farmer entrepreneurship (FEQG) is also found to have a significant and positive effects on rural poverty (RP), and this is in line with the findings of Li et al. [74]. However, the effect of attitude towards farmer entrepreneurship growth (ATFE) on rural poverty (RP) is negative, although it is significant.

The study also shows that about 85% of those actively engaged in farmer entrepreneurship are aged 16–55 years old. This is an indication of existing entrepreneurial opportunities in the rural areas of China for employment in agriculture, which can help reduce youth unemployment. In conclusion, there is a significant relationship between farmer entrepreneurship and rural poverty alleviation in the Guangxi Province of China.

A main policy implication of this study is that the government of China in new attempts to alleviate rural poverty should put more emphasis on rural policies, such as the promotion of farmer entrepreneurship in rural areas. This is because farmer entrepreneurship is a bottom-up and more sustainable approach towards alleviating rural poverty. Furthermore, an approach which comes more from farmers’ own initiative can help to reduce the financial burden of the central government in the form of resource allocation for targeted poverty alleviation in the country.

There are some limitations and future research directions for this study. First, part of the survey was conducted based on self-reported answers from respondents. It is possible there could be some questions that were not clearly understood and well answered due to challenges encountered during the back-and-forth translation of the questionnaire from English to Chinese. Although care was taken to minimize the occurrence of errors, we suggest that more local people in the study areas should be recruited as data enumerators. Second, in addition to the use of the structural equation modeling, different methods of analysis should be employed to ensure greater robustness of the results. Third, in spite of the confidence in the findings of the study, the data were only taken from three local communities in the Guangxi Province of China. In the future, data should be taken from other rural communities to obtain provincial sample representativeness in order to reach a generalized conclusion on the relationship between farmer entrepreneurship and rural poverty alleviation in China.

## Supporting information

S1 Dataset Research data. (XLSX)

S1 Table Survey questionnaire. (PDF)

S2 Table Survey communities and retrieval rate. (PDF)

S3 Table Correlation matrix results. (PDF)
