# Supplementary material for: Does farmer entrepreneurship alleviate rural poverty in China? Evidence from Guangxi Province
Source: PLoS One. 2018 Mar 29;13(3):e0194912. doi: 10.1371/journal.pone.0194912 (PMC5875809; doi:10.1371/journal.pone.0194912)
Supplement: S1 Table — (PDF) [file pone.0194912.s002.pdf]

S1 Table

Item	Definition	Adapted from
b1	More farmers are becoming entrepreneurs	Sen (2000), Granovetter (1985)
b2	More enterprises are being founded by villagers	
b3	Enterprises founded offer more and funds to support development of the village	
b4	Enterprises founded by villagers are getting better every time	
b5	More children now go to school	
b6	Children's education condition is getting better	
b7	The education children receive is increasingly high	
b8	I have more access to high market knowledge/information	
b9	I have access to more knowledge in technology	Nussbaum (2011), Diener et al. (1985)
b10	I have access to more knowledge in management	
b11	I have the opportunity to receive more training	
b12	The medical facilities in village are getting better	
b13	The medical attention level in village is getting higher all the time	
b14	I have more chances to make more money in the village	
b15	I feel that the family income is getting higher than before	
b16	Village management affairs are becoming more open and transparent	Aryee et al. (2002)
b17	Village decision-making affairs are becoming more democratic	
b18	Community members are increasingly willing to express ideas on village affairs.	
b19	Villagers' leisure activities are becoming more abundant	
