# Supplementary material for: Does farmer entrepreneurship alleviate rural poverty in China? Evidence from Guangxi Province
Source: PLoS One. 2018 Mar 29;13(3):e0194912. doi: 10.1371/journal.pone.0194912 (PMC5875809; doi:10.1371/journal.pone.0194912)
Supplement: S2 Table — (PDF) [file pone.0194912.s003.pdf]

S2 Table

Province	City	Village/Town	Total questionnaires administered	Total questionnaires retrieved	Retrieval rate
Guangxi	Baise	Dongyu<br>Huarun Hope<br>Tangxiong<br>Naping<br>Napo<br>Liufeng<br>Layuan<br>Yangyu	356	309	86%
	Liuzhou	Tianyang<br>Xi wangxiao<br>Huarun<br>SanjiangDanzhoudao<br>Danzhouguzhe<br>Danzhou Ancient Town			
	Guilin	Hongyan<br>Gongcheng			

Source: Authors' field survey, 2015.
