# Supplementary material for: Does farmer entrepreneurship alleviate rural poverty in China? Evidence from Guangxi Province
Source: PLoS One. 2018 Mar 29;13(3):e0194912. doi: 10.1371/journal.pone.0194912 (PMC5875809; doi:10.1371/journal.pone.0194912)
Supplement: S3 Table — (PDF) [file pone.0194912.s004.pdf]

S3 Table

Item	Construct	1	2	3	4	5	6	7	8	9	10	16	17	18	12	13	15	19
b1	ATFE	1.00																
b2	ATFE	0.83	1.00															
b3	FEQG	0.46	0.48	1.00														
b4	FEQG	0.50	0.54	0.55	1.00													
b5	SCC	0.12	0.09	0.13	0.22	1.00												
b6	SCC	0.16	0.19	0.18	0.22	0.57	1.00											
b7	SCC	0.03	0.05	0.19	0.20	0.50	0.66	1.00										
b8	EC	0.13	0.08	0.37	0.30	0.18	0.27	0.31	1.00									
b9	EC	0.16	0.12	0.42	0.42	0.22	0.37	0.50	0.73	1.00								
b10	EC	0.13	0.07	0.39	0.38	0.20	0.30	0.39	0.73	0.72	1.00							
b16	EKC	0.14	0.20	0.27	0.36	0.35	0.34	0.31	0.35	0.37	0.31	1.00						
b17	EKC	0.12	0.17	0.28	0.33	0.27	0.39	0.35	0.36	0.39	0.33	0.81	1.00					
b18	EKC	0.12	0.17	0.31	0.34	0.27	0.37	0.38	0.33	0.43	0.26	0.52	0.55	1.00				
b12	RP	0.01	0.13	0.23	0.28	0.31	0.44	0.38	0.29	0.34	0.22	0.36	0.39	0.38	1.00			
b13	RP	0.10	0.22	0.31	0.36	0.31	0.40	0.37	0.38	0.40	0.35	0.41	0.44	0.33	0.81	1.00		
b15	RP	0.26	0.31	0.32	0.41	0.23	0.33	0.35	0.22	0.34	0.21	0.33	0.39	0.39	0.52	0.48	1.00	
b19	RP	0.30	0.36	0.35	0.36	0.13	0.19	0.26	0.24	0.36	0.26	0.32	0.33	0.38	0.49	0.48	0.47	1.00
